# Vascular and Non-HLA autoantibody profiles in hospitalized patients with COVID-19

**DOI:** 10.3389/fimmu.2023.1197326

**Published:** 2023-06-15

**Authors:** Brian Lichtenstein, Ying Zheng, David Gjertson, Kathie G. Ferbas, Anne W. Rimoin, Otto O. Yang, Grace M. Aldrovandi, Joanna M. Schaenman, Elaine F. Reed, Jennifer A. Fulcher

**Affiliations:** ^1^ Division of Hospital Medicine, Department of Internal Medicine, Sharp Rees-Stealy Medical Group, Sharp Healthcare, San Diego, CA, United States; ^2^ Department of Pathology and Laboratory Medicine, David Geffen School of Medicine at UCLA, Los Angeles, CA, United States; ^3^ Department of Biostatistics, Fielding School of Public Health, University of California Los Angeles, Los Angeles, CA, United States; ^4^ Division of Infectious Diseases, Department of Pediatrics, David Geffen School of Medicine at UCLA, Los Angeles, CA, United States; ^5^ Department of Epidemiology, Fielding School of Public Health, University of California Los Angeles, Los Angeles, CA, United States; ^6^ Division of Infectious Diseases, Department of Medicine, David Geffen School of Medicine at UCLA, Los Angeles, CA, United States; ^7^ Infectious Diseases Section, Veterans Affairs (VA) Greater Los Angeles Healthcare System, Los Angeles, CA, United States

**Keywords:** COVID-19, autoantibody, angiotensin II receptor type 1 (AT1R), Non-HLA antigens, Anti-endothelial antibodies

## Abstract

**Introduction:**

Severe COVID-19 illness is characterized by an overwhelming immune hyperactivation. Autoantibodies against vascular, tissue, and cytokine antigens have been detected across the spectrum of COVID-19. How these autoantibodies correlate with COVID-19 severity is not fully defined.

**Methods:**

We performed an exploratory study to investigate the expression of vascular and non-HLA autoantibodies in 110 hospitalized patients with COVID-19 ranging from moderate to critically ill. Relationships between autoantibodies and COVID- 19 severity and clinical risk factors were examined using logistic regression analysis.

**Results:**

There were no absolute differences in levels of expression of autoantibodies against angiotensin II receptor type 1 (AT1R) or endothelial cell proteins between COVID-19 severity groups. AT1R autoantibody expression also did not differ by age, sex, or diabetes status. Using a multiplex panel of 60 non- HLA autoantigens we did identify seven autoantibodies that differed by COVID-19 severity including myosin (myosin; p=0.02), SHC-transforming protein 3 (shc3; p=0.07), peroxisome proliferator-activated receptor gamma coactivator 1-beta (perc; p=0.05), glial-cell derived neurotrophic factor (gdnf; p=0.07), enolase 1 (eno1; p=0.08), latrophilin-1 (lphn1; p=0.08), and collagen VI (coll6; p=0.05) with greater breadth and higher expression levels seen in less severe COVID-19.

**Discussion:**

Overall, we found that patients hospitalized with COVID-19 demonstrate evidence of auto-reactive antibodies targeting endothelial cells, angiotensin II receptors, and numerous structural proteins including collagens. Phenotypic severity did not correlate with specific autoantibodies. This exploratory study underscores the importance of better understanding of the role of autoimmunity in COVID-19 disease and sequelae.

## 1 Introduction

While SARS-CoV-2 infection causes mild illness in most persons, a minority develop severe COVID-19 that can progress to acute respiratory distress syndrome, multiorgan failure, and death. Severe COVID-19 is characterized by an overwhelming immune response with elevated pro-inflammatory cytokines and innate immune hyperactivation (1, 2). Current pharmacotherapy for COVID-19 targets these immune mechanisms *via* the routine use immunomodulatory therapy (glucocorticoids, IL6 inhibitors and JAK inhibitors in severe to critical cases of COVID-19) (3). In the case of severe COVID-19, the wide spectrum of illness and multiorgan involvement may also be related to the presence and severity of thrombovasculitic disease. Indeed, two post-mortem studies demonstrated that microthrombotic angiopathy and endothelialitis in major organs are the predominant pathologic findings of patients who die due to overwhelming COVID-19 (4, 5).

Autoimmunity against the renin-angiotensin system, which regulates vascular tone, is a well-established pathology in vasculitic disease in humans. Auto-antibodies against angiotensin converting enzyme 2 (ACE2) and endothelial cell proteins are known to correlate with both the presence and severity of vasculitis diseases including systemic lupus erythematosus, anti-phospholipid syndrome, rheumatoid arthritis, systemic sclerosis, and Kawasaki disease (6–9). Angiotensin II receptor type 1 auto-antibodies (AT1R-Ab) represent another source of immune pathology in humans. Organ transplant patients who develop HLA-negative antibody mediated rejection typically do so as a result of circulating AT1R-Ab against the allograft tissue (10–15). These antibodies appear to trigger vascular inflammation in transplanted organs that is accompanied by the production of inflammatory cytokines with associated graft vasculopathy and allograft dysfunction (16, 17). Auto-immunity against endothelial cell proteins may also be a factor in such cases, as the presence of AT1R-Ab also correlates strongly with level of anti-endothelial cell antibodies (AECA) as measured by the endothelial cell flow cytometric crossmatch (ECXM) assay (12, 18). Perhaps it comes as little surprise that patients with COVID-19 also express these autoimmune markers. Among patients with severe or unfavorable courses with COVID-19, AT1R-Ab and anti-endothelial antibodies were higher than those with mild COVID-19 and/or matched controls (19, 20). However, other studies found that patients with mild COVID-19 had higher AT1R compared to patients with more severe COVID-19 and/or healthy controls (21, 22). Severe COVID-19 can also induce anti-angiotensin II antibodies which correlated with poor oxygenation and blood pressure dysregulation in these patients (23).

Understanding the breadth and magnitude of autoantibody responses across COVID-19 will be important to better understand pathophysiology of this disease and its sequelae. In addition to vascular antigens, autoantibodies against tissue and cytokine antigens develop during SARS-CoV-2 infection in hospitalized patients (24, 25), and track with the onset of SARS-CoV-2 immune responses (25). Persistent autoantibody responses have also been found in recovered patients following mild or asymptomatic SARS-CoV-2 infection (26). While specific autoantibodies against vascular and cytokine antigens have been correlated with COVID-19 clinical severity (24, 25, 27, 28), the relationships between autoantibody breadth and clinical outcomes have not been established. Here we investigated not only the prevalence of vascular autoimmune responses, but also further examined the breadth of the autoantibody response including other tissue antigens as correlates of phenotypic severity in patients hospitalized with COVID-19. We hypothesized that greater autoimmune responses would correlate with more severe COVID-19 disease.

## 2 Materials and methods

### 2.1 Ethics statement

The study was approved by the UCLA Institutional Review Board (#20-000473) and the Sharp Healthcare Institutional Review Board (#2012806). Informed consent was obtained from all study participants.

### 2.2 Study participants

Patients were recruited at two large healthcare organizations in Southern California. Inclusion criteria included hospitalization for COVID-19, age greater than 18 years, and confirmed positive SARS-CoV-2 RT-PCR test results within 72 hours of admission.

UCLA Health is a large academic healthcare organization in Los Angeles, California. Patients with confirmed positive SARS-CoV-2 RT-PCR nasopharyngeal swab were enrolled in an observational cohort study within 72 hours of admission. Exclusion criteria included pregnancy, hemoglobin < 8g/dL, or inability to provide informed consent. Blood specimens, nasopharyngeal swab, and saliva were collected throughout hospitalization up to 6 weeks. A total of 80 patients from UCLA Health were included in this study from April 2020 until February 2021. Samples from the first study visit after admission (within 72 hours) were used in this analysis.

Sharp HealthCare is a regional healthcare organization in San Diego, California. Patients with confirmed positive SARS-CoV-2 RT-PCR nasopharyngeal swab within 72 hours of admission or the seven days preceding admission for hospital transfer were enrolled in this observational study. Exclusion criteria included: prior vaccination against SARS-CoV-2, treatment with anti-CD20 or anti-CD19 monoclonal antibodies, COVID convalescent plasma (CCP) or fresh frozen plasma, IVIG, plasmapheresis, anti-neoplastic chemotherapy, or other biologic or immunosuppressive agents (aside from corticosteroids, IL6 receptor inhibitors, or baricitinib given for treatment of COVID-19) in the three months prior to enrollment, history of organ transplant, or clinically active autoimmune disease. A total of 32 patients from Sharp HealthCare were included in this study from February 2021 until August 2021. Samples from the first study visit after admission (within 72 hours) were used in this analysis. Two enrolled patients were diagnosed with multisystem inflammatory syndrome in adults (MIS-A) and excluded from this analysis, but clinical data for these cases is included in **Supplementary Table 1**.

Demographic and clinical data, including laboratory results and therapeutics, were collected from the electronic medical records. Clinical severity was scored using the NIAID 8-point ordinal scale (29): 1, not hospitalized and no limitations; 2, not hospitalized but with limitations; 3, hospitalized no supplemental oxygen or ongoing medical care; 4, hospitalized no supplemental oxygen but with ongoing medical care; 5, hospitalized with supplemental oxygen; 6, hospitalized with non-invasive ventilation or high-flow oxygen; 7, hospitalized with invasive mechanical ventilation or ECMO; 8, death.

### 2.3 AT1R-Ab quantification

Plasma samples at the first study visit after admission were used for all assays. AT1R-Ab were quantified by ELISA (Cell Trend, Germany) as previously described (17). Briefly, sera were diluted 1:100, tested in duplicate, and AT1R-Ab concentrations were determined by a standard curve. AT1R-Abs exceeding the limit of the standard curve were further diluted and re-tested to determine concentration. AT1R-Ab IgG >10 units/ml was considered at risk for endothelial dysfunction based on laboratory validated manufacturer’s recommended cutoff value.

### 2.4 Endothelial cell crossmatch

Anti-endothelial cell antibodies were quantified using endothelial cell crossmatch by flow cytometry as previously described (30). Briefly, primary human aortic endothelial cells were isolated from aortic rings of explanted donor hearts or obtained from commercial sources. The cells were cultured and passages 7 to 8 were frozen and used in the endothelial cell crossmatch. A total of 2x10^5^ cells were incubated with 100 μl patient serum on ice for 30 minutes, washed and incubated with F(ab′)_2_ fragment Goat Anti-Human IgG Fcγ fragment (Jackson ImmunoResearch Laboratories). Cell fluorescence was analyzed on a FACSCalibur flow cytometer using CellQuest Software (BD Biosciences) with a minimum of 10,000 gated events acquired. The positive endothelial cell crossmatch threshold was set at two standard deviations (50 Median Channel Shift) above the mean of negative control serum tests

### 2.5 Autoantibody quantification by luminex

A custom Luminex panel was used to quantify 60 different autoantibodies as previously described (31). The non-HLA multiplex bead panel was purchased from Immucor, Inc, and included 60 non-HLA antigens conjugated to polystyrene beads (LIFECODES Non-HLA Antibody Kit; cat # 265500R). Forty µL of antigen-coated beads were incubated with 10 µL of serum for 30 minutes. After washing beads were stained with 50 µL of phycoerythrin (PE) conjugated goat anti-human IgG diluted 1:10 in buffer and incubated in the dark on a shaking platform for 30 minutes. Antibody binding was reported as the median fluorescence intensity (MFI) of IgG binding on the Luminex 100 (Luminex). Antibodies with mean MFI >500 in either group were selected for further analysis based on manufacturer’s provided average background expression in healthy control populations resulting in n=36 antibodies. From there a stringent MFI cutoff of 1000 was used to determined positive/negative based on overall assay variance.

### 2.6 Statistical analysis

Autoantibody MFI levels were log-transformed to reduce positive skew of the raw data. Comparisons of mean and median log-MFI estimates were performed between COVID-19 severity groups (moderate/severe vs critical/deceased) using t-tests and Wilcoxon rank sum tests. Comparisons of positive autoantibodies by severity groups were performed using t-tests and Fisher’s exact tests. Associations with COVID-19 risk factors were analyzed using multiple logistic regression. In this exploratory analysis an unadjusted p-value < 0.1 was considered significant. Statistical analyses were conducted using STATA version 17.0 and R version 4.2.1.

## 3 Results

### 3.1 Study population

This study included a total of 110 patients with n=80 patients from the Los Angeles site and n=30 patients from the San Diego site. This was a diverse cohort with 52% Hispanic, 10% Asian/Non-Hispanic, 7% Black/Non-Hispanic, and 20% White/Non-Hispanic. Consistent with COVID-19 hospitalization trends, there were more men (59%) than women. The average age across the study population was 52.5 years. Patients were grouped based on maximum clinical severity during hospitalization as classified by the NIAID 8-point ordinal scale (29), and further combined into moderate to severe (ordinal scale groups 4-5) group or critical to decreased (ordinal scale groups 6-8) group. Patients with moderate to severe disease were on average younger (mean age 49 years) than those with critical to fatal disease (mean age 56 years) (**Table 1**). Comorbidities included cardiovascular disease (57%) and diabetes (68%) in the majority of patients, with greater prevalence of diabetes in the moderate to severe group (78% vs 56%). In addition, this study included 21 patients (19%) with solid organ transplant at the time of study entry. COVID-directed therapies varied based on standard of care at each site and a stage of the pandemic.

**Table 1:** Demographics and clinical characteristics of study population.

	COVID-19 Severity		p-value^a^
	Moderate-Severe n=60	Critical-Deceased n=50	
Demographics			
**Age** (yrs), mean (SD)	49 (14.6)	56 (13.1)	0.01
**Sex** (n, %)			0.85
Male	36 (60%)	29 (58%)	
Female	24 (40%)	21 (42%)	
**Ethnicity** (n, %)			0.01
Hispanic	24 (40%)	33 (66%)	
Non-Hispanic	34 (57%)	17 (34%)	
Declined to state	2 (3%)	0	
**Race** (n, %)			0.16
Asian	6 (10%)	5 (10%)	
Black	6 (10%)	2 (4%)	
White	15 (35%)	7 (14%)	
Other	30 (50%)	36 (72%)	
Declined to state	3 (5%)	0	
Medical History			
**Body mass index (BMI)** (kg/m2), mean (SD)	33 (7.6)	31 (7.4)	0.38
Cardiovascular disease^b^ (n, %)			0.57
Yes	36 (60%)	27 (54%)	
No	24 (40%)	23 (46%)	
**Diabetes** (n, %)			0.01
Yes	47 (78%)	28 (56%)	
No	13 (22%)	22 (44%)	
Pulmonary disease^c^ (n, %)			0.44
Yes	8 (13%)	10 (20%)	
No	52 (87%)	40 (80%)	
Renal disease^d^ (n, %)			0.78
Yes	7 (12%)	7 (14%)	
No	53 (88%)	43 (86%)	
Solid organ transplant^e^ (n, %)			0.48
Yes	13 (22%)	8 (16%)	
No	47 (78%)	42 (84%)	
COVID-19 treatment			
COVID-19 severity score (n, %)			
Hospitalized, no supplemental oxygen (ordinal scale 4)	8 (13%)	0	
Hospitalized, with supplemental oxygen (ordinal scale 5)	52 (87%)	0	
ICU, not ventilated (ordinal scale 6)	0	24 (48%)	
ICU, mechanical ventilation (ordinal scale 7)	0	18 (36%)	
Deceased (ordinal scale 8)	0	8 (16%)	
**Steroids (dexamethasone)** (n, %)			0.003
Yes	36 (60%)	43 (86%)	
No	24 (40%)	7 (14%)	
**Remdesivir** (n, %)			0.03
Yes	39 (65%)	42 (84%)	
No	21 (35%)	8 (16%)	
**Convalescent plasma** (n, %)			<0.001
Yes	4 (7%)	20 (40%)	
No	56 (93%)	30 (60%)	
**Clinical trial** (n, %)			<0.001
Yes	10 (17%)	25 (50%)	
No	50 (83%)	25 (50%)	
**Study Center** (n, %)			0.005
UCLA Health	37 (62%)	43 (86%)	
Sharp Healthcare	23 (38%)	7 (14%)	

a p-values calculated using t-tests for continuous variables and Fisher’s exact test or Chi-square for categorical variables.

b Cardiovascular disease includes hypertension, coronary artery disease, heart failure.

c Pulmonary disease includes asthma, COPD.

d Renal disease includes chronic kidney disease, hemodialysis.

e Solid organ transplant includes kidney transplant, lung transplant, heart transplant, liver transplant.

### 3.2 Prevalence of AT1R-Ab and endothelial cross-reactivity

**Figure 1:**
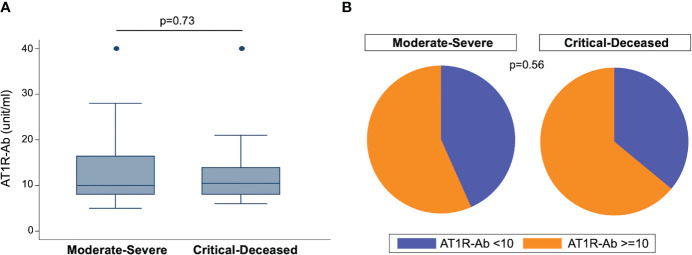
Angiotensin II receptor type 1 antibody (AT1R-Ab) quantification by COVID-19 severity. **(A)** Amount of AT1R-Ab measured by ELISA in plasma collected within 72 hours of hospital admission from patients with COVID-19 grouped by severity. Boxes show the interquartile range (IQR) with the line at the median and whiskers extend to 1.5 times the IQR. Data points outside the range (outliers) are shown with dots. P-value calculated using Wilcoxon rank-sum test. **(B)** Proportion of patients at risk for endothelial cell dysfunction (AT1R-Ab >=10) grouped by COVID-19 severity. P-value calculated using Fisher’s exact test.

To assess the prevalence of ATR1-Ab in COVID-19 patients we used ELISA to quantify anti-AT1R IgG in plasma samples collected within 72 hours of hospital admission. The median concentration of AT1R-Ab was similar across both severity groups (**Figure 1A**). However, when looking at the proportion of patients with ATR1-Ab levels above the threshold for endothelial dysfunction risk (>10 U/ml), the majority of patients in both severity groups had elevated ATR1-Ab with the critically ill group having slightly greater proportion (**Figure 1B**).

**Figure 2:**
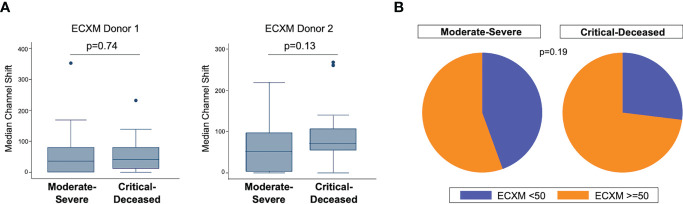
Anti-endothelial cell antibodies by COVID-19 severity. **(A)** Anti-endothelial cell antibodies were measured in plasma collected within 72 hours of hospital admission by endothelial cell crossmatch using two separate donors shown as median channel shift above negative control serum. Boxes show the interquartile range (IQR) with the line at the median and whiskers extend to 1.5 times the IQR. Data points outside the range (outliers) are shown with dots. P-values calculated using Wilcoxon rank-sum tests. **(B)** Proportion of patients with positive endothelial cell crossmatch (EXCM >=50 based on two standard deviations above negative control serum) grouped by COVID-19 severity. P-value calculated using Fisher’s exact test.

We also examined endothelial cell cross-reactivity using a flow cytometry-based method to examine presence of cross-reactive antibodies against human aortic endothelial cells (ECXM) (30). The median ECXM scores were higher in the critically ill group but did not meet statistical significance (**Figure 2A**). When looking at the proportion of patients in each clinical group with ECXM greater than 50 median channel shift, again the majority of patients in both groups had evidence of anti-endothelial cell antibodies with the critically ill having the greatest proportion (**Figure 2B**; p=0.19).

We next used regression analysis to further examine associations between AT1R-Ab and COVID-19 severity. There was no significant association between AT1R-Ab and COVID-19 severity in univariate analysis (OR 1.02, 95% CI [0.97, 1.07], p=0.45). We still found no significant associations with AT1R-Ab using multiple logistic regression including age, sex, ethnicity, race, BMI, diabetes, cardiovascular disease, pulmonary disease, kidney disease, or transplant status. We again used regression analysis to further examine associations between ECXM and COVID-19 severity, but found no significant association between ECXM and COVID-19 severity in univariate analysis (OR 2.17, 95% CI [0.73, 6.44], p=0.16) or multivariate analysis.

### 3.3 Correlation of vascular autoantibodies and COVID-19 risk factors

**Figure 3:**
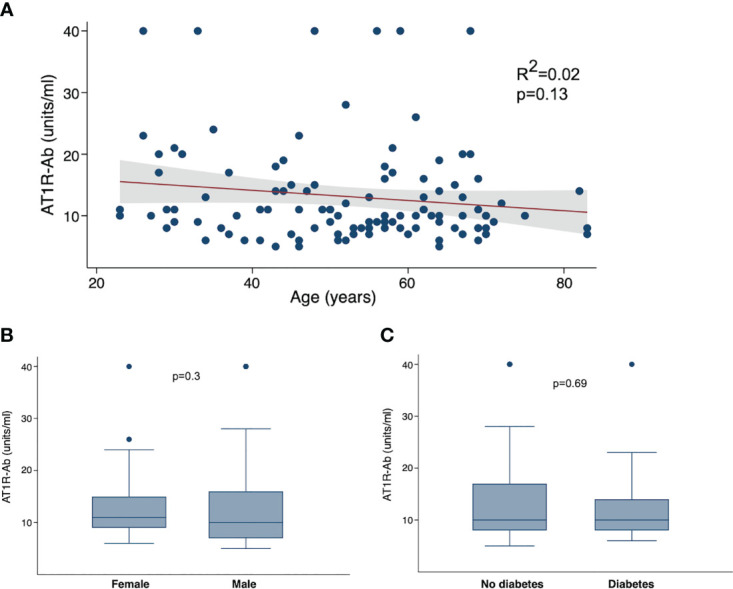
Angiotensin II receptor type 1 antibody (AT1R-Ab) by COVID-19 clinical characteristics. **(A)** AT1R-Ab quantification plotted by age in years. P-value and R^2^ calculated using linear regression. AT1R-Ab quantification by **(B)** sex and **(C)** diabetes status. Boxes show the interquartile range (IQR) with the line at the median and whiskers extend to 1.5 times the IQR. Data points outside the range (outliers) are shown with dots. P-values calculated using Wilcoxon rank sum tests.

We next examined the correlations between AT1R autoantibodies and known COVID-19 risk factors. As the presence of autoantibodies can increase with age (32), we hypothesized that the presence of autoantibodies may be a mediating factor in the association between COVID-19 severity and age. However, we found no correlation between age and ATR1-Ab (**Figure 3A**). We also looked at comparisons of autoantibodies by sex, but again found no specific difference of AT1R-Ab by sex (**Figure 3B**). Since diabetes is also a significant risk factor for severe COVID-19 we compared autoantibodies by diabetes status and found no significant difference in AT1R-Ab in those with or without diabetes (**Figure 3C**).

### 3.4 Expression of non-HLA self-reactive antibodies in COVID-19

**Figure 4:**
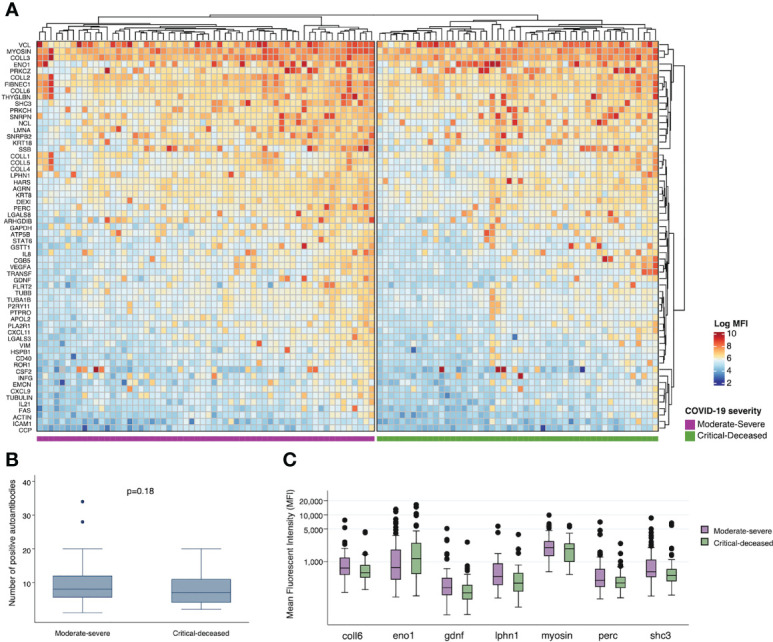
Plasma autoantibodies by COVID-19 severity. **(A)** Heatmap showing relative expression of autoantibodies in plasma collected within 72 hours of hospital admission among moderate-severe (left) or critical-deceased (right) COVID-19 patients. **(B)** Comparison of number of positive autoantibodies by COVID-19 severity. P-value calculated using t-test. **(C)** Relative expression of plasma autoantibodies compared by COVID-19 severity (orange, moderate-severe; blue, critical-deceased). Only antibodies with p<=0.1 by Fisher’s exact tests are shown. Boxes show the interquartile range (IQR) with the line at the median and whiskers extend to 1.5 times the IQR. Data points outside the range (outliers) are shown with dots.

To assess for other targets of autoimmunity, we used a multiplex panel to quantify self-reactive antibodies against 60 collagen-vascular antigens. We used unsupervised hierarchical clustering to visualize differences in these autoantibodies by COVID-19 severity. Comparing each severity group there does appear to be overall higher expression of autoantibodies in the moderate-severe compared to the critical-deceased group (**Figure 4A**), but the number of positive autoantibodies did not significantly differ by severity groups (p=0.18; **Figure 4B**). The autoantibodies with the most significant difference in positivity between severity groups included myosin (myosin; p=0.02), SHC-transforming protein 3 (shc3; p=0.07), peroxisome proliferator-activated receptor gamma coactivator 1-beta (perc; p=0.05), glial-cell derived neurotrophic factor (gdnf; p=0.07), enolase 1 (eno1; p=0.08), latrophilin-1 (lphn1; p=0.08), and collagen VI (coll6; p=0.05). With the exception of enolase 1, expression of these autoantibodies was higher in the moderate-severe group compared to the critical-deceased (**Supplementary Table 2** and **Figure 4C**).

## 4 Discussion

Mounting evidence implicates a dysregulated host immune response in the pathophysiology of COVID-19. Severe disease typically presents as a rapid progression of worsening dyspnea and hypoxemia around the second week following infection, a time course is also coincident with the early humoral immune response (33–35). The presence of autoantibodies has been described in several clinical studies resulting in a need to better understand the potential role of autoantibodies in contributing to COVID-19 disease. This exploratory study sought to expand this understanding, specifically examining the presence of autoantibodies against endothelial cells and collagen-vascular tissue. We did not find an association with AT1R-Ab or AECA and COVID-19 severity. We did identify the presence of other collagen-vascular autoantibodies in hospitalized patients with COVID-19, and those with less severe COVID-19 actually had higher autoantibody levels.

Our data are consistent with prior studies detailing the presence of autoantibodies in COVID-19. While others showed associations between AT1R and COVID-19 severity (19, 20), we did not find similar associations. At least one other study also found no difference in AT1R-Ab when comparing ICU patients and non-ICU hospitalized patients (22). Several factors may contribute to these differences. The timing of sampling during disease course may differ between studies which could affect the ability to detect autoantibodies. Though several studies, including ours, controlled for comorbid conditions or other confounding factors, there is still heterogeneity between study populations as well as differences in COVID-directed therapeutics that may affect results. The totality of these studies suggest that these autoantibodies may play a role in COVID-19, and further investigation with larger longitudinal analysis will be necessary to improve understanding.

Virus-triggered immune dysregulation contributes not only to the acute presentation, but also to post-infection sequelae. Well known pathogens such as enteroviruses and Parvovirus B19 are associated with and potentially causative of autoimmune diseases including rheumatoid arthritis and type 1 diabetes mellitus (36, 37). More recently, prior infection with Epstein-Barr virus was shown to increase odds of later developing multiple sclerosis (38). Many of these same autoimmune epitopes observed in our study are also implicated in the development and progression of autoimmune disease. Anti-collagen antibodies have been observed in the early phase response of rheumatoid arthritis (39) and anti-enolase in a multitude of autoimmune diseases (40). Similar collagen autoantibodies have also been implicated in transplant allograft rejection (41). It is possible that the severe immune activation that characterizes COVID-19 leads to either epitope spread or bystander activation resulting in loss of self-tolerance and autoantibody production. Indeed, there have been reports describing increased incidence of autoimmune disease following COVID-19 (42–44). Interestingly, there have been reports of higher risk of new onset autoimmune disease following mild to moderate COVID-19 (43); we also observed greater autoantibody levels in the moderate group compared to critical. Longitudinal studies will be important to determine if COVID-19 severity and/or extent of autoantibody responses during COVID-19 are risk factors for subsequent autoimmune disease.

Our study has several important limitations to consider when interpreting the data. We did not have non-COVID-19 control samples available for analysis, thus can only draw conclusions within our COVID-19 severity groups. While we can compare our results to historical controls and published studies from before SARS-CoV-2, the lack of a within study control group is a limitation. Our study population also had heterogeneity in terms of treatments received and comorbidities, including solid organ transplantation. We accounted for these differences in our analyses as able, but these may still confound our results. Finally, our study is cross-sectional which limits any conclusions regarding causality between autoantibody presence and COVID-19 outcomes or future disease risk.

Patients hospitalized with COVID-19 demonstrated evidence of auto-reactive antibodies targeting endothelial cells, angiotensin II receptors, and numerous structural proteins including collagens. In this exploratory study, phenotypic severity did not correlate with specific autoantibodies. Further research is required to understand the mechanisms and clinical implications of autoimmunity following infection with SARS-CoV-2.

## Data availability statement

The original contributions presented in the study are included in the article/**Supplementary Material**. Further inquiries can be directed to the corresponding author.

## Ethics statement

The studies involving human participants were reviewed and approved by UCLA Institutional Review Board (#20-000473) and Sharp Healthcare Institutional Review Board (#2012806). The patients/participants provided their written informed consent to participate in this study.

## Author contributions

BL and JF conceived of the study and BL, JF, and ER designed the study. KF, AR, OY, GA contributed to overall cohort design, sample processing, and funding support. JS contributed to study recruitment and implementation. YZ, DG, and JF performed analyses. BL, JF, and ER wrote the manuscript. All authors contributed to the article and approved the submitted version.
